# Effects of Incretin Pathway Elements on Bone Properties

**DOI:** 10.7759/cureus.33656

**Published:** 2023-01-11

**Authors:** Arezoo M Abdi, Ermioni Pasiou, Panagiotis Konstantopoulos, Tatiana S Driva, Athanasios Kontos, Eleni Papagianni, Stavros Kourkoulis, Dimitrios Dimitroulis, Despoina N Perrea, John Vlamis

**Affiliations:** 1 Laboratory of Experimental Surgery and Surgical Research "N.S. Christeas" (LESSR), National and Kapodistrian University of Athens, Athens, GRC; 2 Department of Mechanics, National Technical University of Athens, Athens, GRC; 3 Laboratory of Experimental Surgery and Surgical Research "N.S. Christeas" (LESSR), National & Kapodistrian University of Athens, Athens, GRC; 4 Department of Physics, National Technical University of Athens, Athens, GRC; 5 Department of Pathology, Evangelismos General Hospital, Athens, GRC; 6 Laboratory of Experimental Surgery and Surgical Research "N.S. Christeas" (LESSR), National and Kapodistrian University of Athens School of Medicine, Athens, GRC; 7 Department of Orthopedics, University of Athens, KAT Hospital, Athens, GRC

**Keywords:** glucose, exenatide, sitagliptin, osteoporosis, bone biomechanics, incretins, diabetes mellitus, type ii diabetes

## Abstract

Introduction

The effects of incretin-based drugs, such as receptor agonists of glucagon-like peptide-1 and inhibitors of dipeptidyl peptidase-4, on bone metabolism are not completely clear yet. The aim of this study is to compare the effects of glucagon-like peptide-1 and inhibitors of dipeptidyl peptidase-4 on the bone to see how different elements of the incretin pathway affect bone quality in terms of biomechanical properties, bone turnover, and mineral properties.

Materials and methods

Forty 10-week-old Wistar rats were divided into four groups: a control group, a control diabetic group, a diabetic group treated with sitagliptin, and a diabetic group treated with exenatide. Type 2 diabetes was simulated by dietary manipulation in addition to low-dose streptozotocin, and then two different incretin-based drugs were administered. The rats were sacrificed after five weeks of therapeutic treatment. Their serum was analyzed with the enzyme-linked immunosorbent assay (ELISA) method for basic bone turnover markers, and their right femur was subjected to a three-point bending test. Finally, Hematoxylin & Eosin staining, in addition to Raman spectroscopy, were employed to access the collagen and mineral properties of the bone.

Results

Both incretin-based drugs reduced osteoclast function; however, they were not able to restore osteoblastic function to normal. The net effect on bone strength was surprising: bone elasticity was restored by the antidiabetic treatment, but bone strength deteriorated. Exenatide had a slightly more pronounced effect, which, although not significant, points to the direction that dipeptidyl peptidase-4 (DPP4) may be a linking factor between reduced osteoclastic function and reduced bone formation, as suggested by the literature.

Conclusion

DPP4 receptors seem to be one of the links between reduced osteoclast function and reduced bone remodeling, so DPP4 inhibition can be more detrimental to the bone than glucagon-like peptide-1 (GLP-1) receptor agonists.

## Introduction

Bone is a tissue with multiple functions: 1) it supports body weight and movement and protects internal organs; 2) it functions as a mineral reservoir for calcium and phosphate; 3) it provides a scaffold for hematopoietic cells; and 4) it participates in multiple endocrine pathways, including fat metabolism, calcium homeostasis, and hormone production [1].

As a part of its multiple functions, bone constantly undergoes cycles of absorption and reconstruction, a process commonly known as bone remodeling. This delicate equilibrium between bone absorption and bone reconstruction is subjected to multiple pathways of regulation which are still under investigation and are linked to the pathogenesis of osteoporosis. Factors that have been known to participate in the regulation of bone homeostasis are, between others, sex hormones, autocrine/ paracrine signals, mechanical loading, as well as metabolic hormones (insulin, glucagon) [2].

Recently, there has been an increased interest in the effect of diabetes on bone turnover. Due to the growing incidence of both types of diabetes, the lesser-known effects, such as diminished bone metabolism, are being studied thoroughly. The net result of diabetes on fracture risk and the result of the treatment options on bone metabolism remain unclarified. It has been shown that diabetes mellitus increases the risk of osteoporotic fractures in the clinical setting; however, not all in vivo studies have managed to replicate the loss of bone strength in diabetic animals [3].

All studies agree that patients with type II diabetes mellitus are more prone to bone fractures; however, their bone mineral density is normal or even increased. This implies multiple factors besides bone quantity involved in total bone fragility, probably bone quality [4-5].

Diabetes has both direct and indirect effects on bone health. Although in vivo and in vitro studies show that diabetes consistently lowers bone formation, results on bone resorption are not as straightforward. Most animal studies suggest increased bone resorption in type 2 diabetes, whereas clinical studies show decreased bone turnover in general [6]. Secondarily, hyperglycemia increases the production of advanced glycation end products (AGEs), which in turn alter the structure of bone collagen fibers resulting in decreased material properties and increased cortical bone porosity [7]. Diabetes is also known to affect the microvascular structure, which contains bone microvasculature, resulting in increased bone marrow adiposity/ skeletal alterations and decreased fracture healing [8].

Incretin hormones are released in the bloodstream from the gastrointestinal tract after glucose absorption and amplify the insulin secretion caused by hyperglycemia. Glucose-dependent insulinotropic polypeptide (GIP) and glucagon-like peptide-1 (GLP-1) are the most studied incretin hormones. The incretin effect is a two- to three-fold higher insulin secretory response to oral as compared to intravenous glucose administration, an effect that is blunted in type 2 diabetes patients. When in circulation, both hormones are rapidly deactivated by an enzyme called dipeptidyl peptidase-4 (DPP-4) [9]. Based on this concept, glucagon-like peptide-1 receptor agonists and dipeptidyl peptidase-4 inhibitors are a novel drug class that is used for type two diabetes and was found to have a lot of extra-pancreatic effects as well.

As far as incretins' relationship to bone metabolism is concerned, many studies have highlighted the effect of gut-secreted hormones on bone turnover. There seems to be a correlation between food fractionation and bone metabolism because distributed calorie consumption during the day has a beneficial effect on bone metabolism compared to one meal a day pattern. In addition, parenteral feeding reduces bone turnover [10]. There are many in vitro and in vivo studies that will be discussed later, exploring the effects of DPP-4 inhibition and GLP-1 agonists in bone cells and overall bone metabolism separately. Both molecules can modulate osteoblast and osteoclast function, but each one has different molecular mechanisms to do so [11].

In this study, we tried to compare the two categories of incretin-based drugs concerning their effect on the mechanical features of bone, on bone mineral composition and on bone cells. Our hypothesis is that if the DPP-4 inhibition and GLP-1 agonists influence independent parts of bone metabolism, their partial effects will differ. The aim of this study was to see if there were any particular differences in the result of the two drugs on bone physiology.

## Materials and methods

Animals and treatment

A total of 40 male 10-week-old Wistar rats (400-450 gr) were purchased from the National Centre for Scientific Research "Demokritos" (Athens, Greece). Animals were housed in pathogen-free conditions (temperature at 20 ± 1 °C, 12-hour light/dark cycles, and 50 ± 5% humidity) and were allowed to adapt to the environment for a week. Then they were randomly divided into four groups: the first group was the control group, the second group was the control diabetic group, the diabetic group treated with sitagliptin was the third group, and the diabetic group treated with subcutaneous exenatide was the fourth group.

A fructose-streptozotocin protocol was used to simulate type 2 diabetes in groups two, three, and four [12]. Specifically, during a three-week induction period, group one received normal drinking water whilst groups two, three, and four received 10% (w/v) fructose in drinking water ad libitum. After three weeks of dietary manipulation, all groups except group 1 received a single intraperitoneal injection of streptozotocin (40 mg/kg b.w.; Sigma-Aldrich, St. Louis, Missouri) dissolved in citrate buffer (pH 4.4, Sigma-Aldrich, St. Louis, Missouri) and group 1 received an intraperitoneal vehicle buffer injection. One week after the injections, animals with non-fasting blood glucose levels greater than 300 mg/dl were considered diabetic (measured with One Touch Verio; LifeScan, Milpitas, California), as suggested by relevant literature [13]. The streptozotocin injection was repeated if necessary. All groups were fed a normal pellet diet during the experiment and were weighed every 10 days.

After confirming diabetes in groups two, three, and four, the third group started oral administration of sitagliptin (Merck & Co., Rahway, New Jersey) in a 10 mg/kg/day dose in their drinking water and group four started receiving weekly subcutaneous injections of extended-release exenatide (AstraZeneca, Cambridge, United Kingdom) (10μg/kg/week). Groups one, two, and three received weekly subcutaneous injections of normal saline at the same time. After five weeks of treatment, all groups were euthanized with cervical dislocation under ether anesthesia.

Biomechanical studies

Animals were euthanized with cervical dislocation facilitated by inhaled anesthetic. Shortly afterward, the right femurs of the rats were removed, cleaned from soft tissues, and transferred to the mechanical testing laboratory to study their mechanical properties in three-point bending testing. 

**Figure 1:**
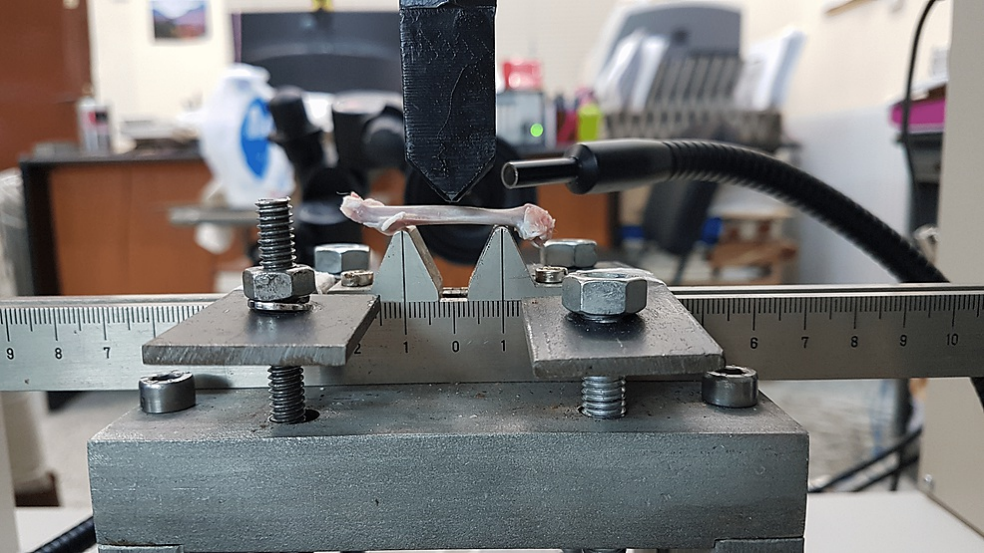
Electromechanical loading frame

Because of the small size of the femora, we used a special frame to support the bones in an MTS electromechanical loading frame (MTS Systems, Eden Prairie, Minnesota) (Figure 1).

The frame provided two supports spaced by 20 mm, and it was kept the same in all experi­ments. The test imposed monotonical displacement at a con­stant rate of 0.5 mm/min on each specimen and was terminated with the fracture of each specimen. A contactless video-extensometer (RTSS, Limess Messtechnik, Krefeld, Germany) filmed each test in order to assess the mid-span deflection of the bones.

To determine the direction of the loading axis, the point of the bone which was in contact with the loading punch was marked red, and the respective lower point of the femur was marked black during the pre-load phase. 

**Figure 2:**
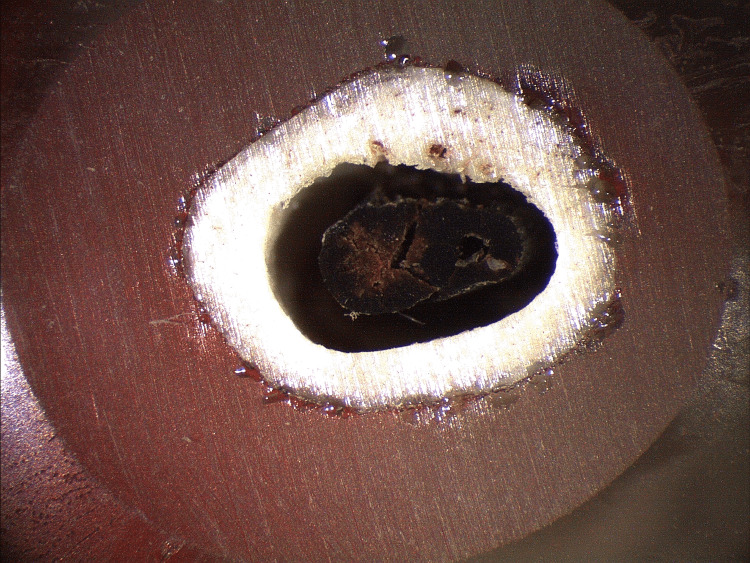
Example of cross-sectional photos of the samples

After the three-point bending tests, one of the two fractured parts of each specimen was fixed vertically in a plastic cup with the use of molten resin. The upper surface of each cup was then smoothed out with a series of abrasive papers to capture the cross-sectional area with a stereoscope (Figure 2).

This is a procedure proposed by Biewener [14] and refined by Kourkoulis et al. [15] that helps us take into account all geometrical characteristics of the cross-sectional area. The data from the cross sections was used to consider the actual shape of the bone and the angle between the loading axis and the central axis of each specimen to improve accuracy.

Raman spectroscopy

After identifying the optimum Raman measurement settings for the encapsulated bone samples, three measurements per rat bone sample/ specimen were performed. The spectra were acquired using the Renshaw Windows-based Raman Environment (WiRE) software. Using the same software, the baseline/ sample noise was subtracted from all measurements. Then a curve fitting procedure was performed using a custom 23-curve custom curve template. The height/ area measurements were transferred to Microsoft Excel (Microsoft, Redmond, Washington) and organized into sample groups. Unnecessary information was removed to reduce confusion.

According to Mandair and Morris [16], absolute band intensities are not useful in Raman spectroscopy because they are heavily skewed by Raman scattering efficiency and other optical effects. Therefore, most studies prefer to use relative peak intensity ratios of defined pairs of bands from each spectrum. 

The most popular Raman spectrum ratios used in bone quality assessment are the mineral-to-matrix ratio, carbonate-to-phosphate ratio, and mineral crystallinity. Mineral-to-matrix ratio can be assessed by the phosphate-to-amide I area ratio and is considered a measure of bone mineralization. Together with the carbonate-to-matrix ratio, they are the strongest predictors of mechanical properties. Carbonate-to-phosphate ratios on the other hand, can provide information about the chemical bone composition that differs with age and mineral crystallinity. Another carbonate-related parameter includes carbonate-to-amide I ratios, which may indicate bone remodeling [17].

We have reported the relative peak intensity ratios of phosphate to CH2 as a mineral-to-matrix ratio, the inverse of the phosphate band as a measure of crystallinity, and the carbonate-to-phosphate ratio as a measure of collagen maturity.

Serum tests

Blood was collected from the retro-orbital sinus at three time points: at the start of the experiment, after the streptozotocin injection, and at the end of the experiment. Osteocalcin was used as a marker of bone formation, tartrate-resistant acid phosphatase 5 (TRAP-5) as a marker of bone resorption, and GLP-1 as a marker of glycemic control. These were evaluated with commercially available rat enzyme-linked immunosorbent assay (ELISA) kits according to the manufacturer's instructions (Glucagon Like Peptide, Rat OC/BGP, Rat ACP5- Tartrate Resistant Acid Phosphatase 5 ELISA kits; Elabscience Biotechnology Co., Wuhan, China).

Histomorphometric analysis 

Left femurs were evaluated histologically after decalcification in 0.5 M ethylenediaminetetraacetic acid (EDTA) solution (pH 7.8) and paraffin embedment. Each specimen was cut in the coronal axis in 5μm thick sections that were stained with hematoxylin and eosin. The sections stained with hematoxylin and eosin were studied, and the number of osteoblasts and osteoclasts per optical view was counted using a light microscope.

Statistical analysis

Data was analyzed using SPSS version 26.0 (IBM Inc., Armonk, New York). Data was analyzed with ANOVA or non-parametric Kruskal-Wallis test, after a z-test to test them for normality. In case of statistical differences, the post hoc Tukey test was used. The p-values less than 0.05 were considered significant.

## Results

Blood sugar

**Figure 3:**
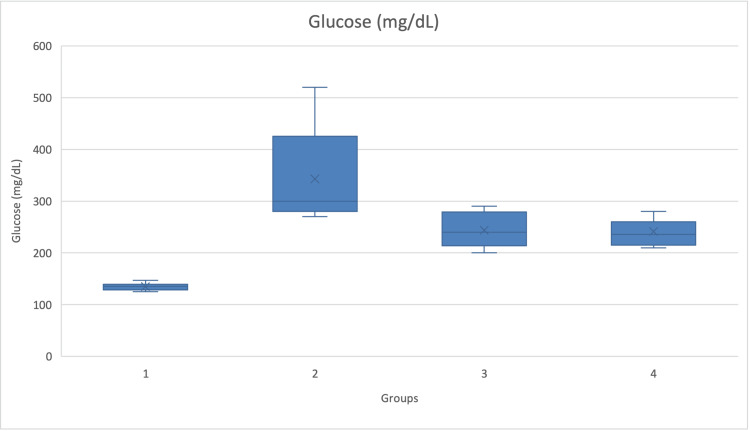
Blood sugar per group (mg/dL) Graphic of blood sugar (mg/dL) and SD in the four different groups at the end of the experiment. Blood sugar was significantly higher in the diabetic group and relatively higher in the diabetic groups treated with incretin-based drugs (p=0.000) compared to the control group.

As seen in Figure 3, blood sugar at the end of the experiment was expectedly significantly higher in the diabetic group. The use of sitagliptin and exenatide partially lowered blood sugar.

Biomechanical studies

**Figure 4:**
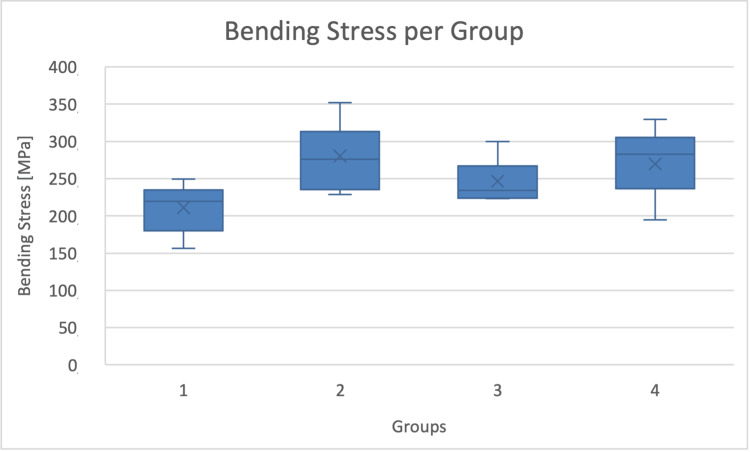
Bending stresses per group Graphic of bending stresses and SD in the four different groups. Bending stresses were significantly higher (p=0.004) in the unmedicated diabetes group. The effect was partially reversed by the antidiabetic treatment.

As seen in Figure 4, bending strength of the diabetic rats was unexpectedly increased compared to the control group. The use of sitagliptin and exenatide partially reverses the effect of diabetes, with exenatide being slightly more "effective".

The same differences can be seen for the equivalent von Mises stresses. This was expected since the shear stresses produced by the torsional moment developed were proven not significant in this protocol (Table 1).

**Table 1:** Biomechanical markers Values are the mean, standard deviation, and one-way ANOVA significance for biomechanical markers.

Group	1		2		3		4		Sig
	Mean	SD	Mean	SD	Mean	SD	Mean	SD	
Bending stress (MPa)	211.04	32.42	280.47	44.63	246.70	26.95	269.56	42.64	0.004
Torsional stress (MPa)	3.71	3.37	8.10	5.82	7.69	5.80	8.71	5.72	0.226
Von Mises stress (MPa)	211.20	32.49	280.94	44.88	247.23	27.10	270.10	42.92	0.004

Raman spectroscopy

**Figure 5:**
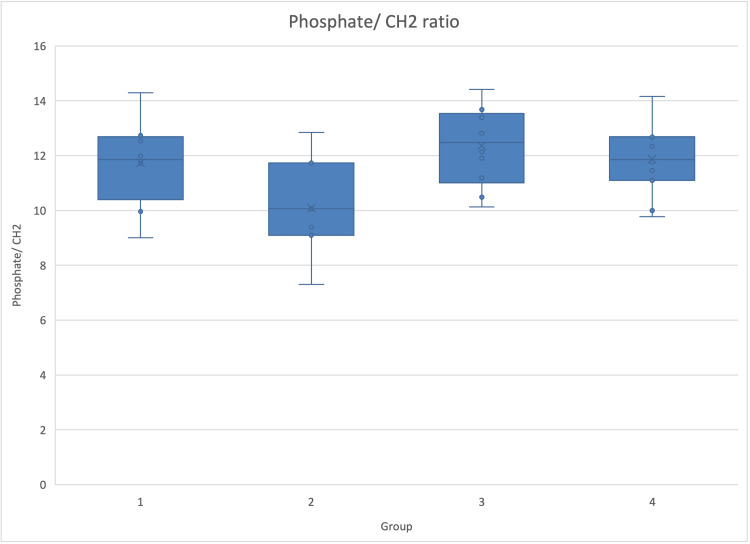
Phosphate-to-CH2 ratio per group Graphic of phosphate-to-CH2 ratio, a marker of mineralization, and SD in the four different groups. The ratio was significantly lower in the untreated diabetic group, implying a less mineralized bone (p=0.032).

The phosphate-to-CH2 ratio was significantly higher in both drug groups, suggesting a higher mineralization rate (Figure 5).

There were no statistically significant differences in phosphate bandwidth and carbonate-to-phosphate ratio (Table 2). 

**Table 2:** Raman spectroscopy ratios Values are mean, standard deviation, and one-way ANOVA significance for Raman spectroscopy ratios studied.

Group	1		2		3		4		Sig
	Mean	SD	Mean	SD	Mean	SD	Mean	SD	
Phosphate/CH2	11.74	1.64	10.08	1.81	12.36	1.44	11.87	1.28	0.032
1/Primary phosphate bandwidth	0.06	0	0.06	0	0.06	0	0.06	0	0.166
Carbonate/phosphate	0.14	0.03	0.14	0.02	0.13	0.02	0.12	0.01	0.271

Serum bone markers/ histomorphometric analysis

**Figure 6:**
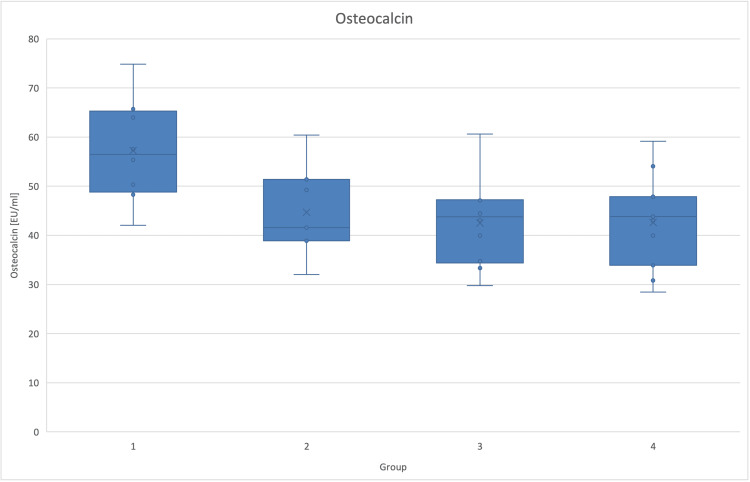
Osteocalcin (EU/ml) per group Graphic of osteocalcin and SD, that was used as a marker of bone formation, in the four different groups. Osteocalcin was significantly lower in all diabetic animals (p=0.008). The effect was not reversed by the antidiabetic treatment.

Following statistical testing, there was less expression of osteocalcin in all diabetic groups compared to the control group (Figure 6).

Conversely, there was no difference in the expression of TRAP-5 and GLP-1 among the groups (Table 3). The differences between osteoblast numbers were not significant either.

**Table 3:** ELISA/histopathology results Values are mean, standard deviation and one-way ANOVA significance for the ELISA method measurements, and histopathology results of osteoblast/ osteoclast numbers. ELISA - enzyme-linked immunosorbent assay, GLP-1 - glucagon-like peptide-1, TRAP-5 - tartrate-resistant acid phosphatase 5

Group	1		2		3		4		Sig
	Mean	SD	Mean	SD	Mean	SD	Mean	SD	
GLP-1 (EU/ml)	1923.39	188.16	1897.73	153.18	1996.65	108.24	1862.91	204.01	0.344
TRAP-5 (EU/ml)	1.06	0.70	0.79	0.51	0.83	0.74	0.63	0.38	0.501
Osteocalcin (EU/ml)	57.27	10.62	44.69	9.53	42.53	8.81	42.63	9.27	0.008
Osteoclasts	3.36	1.38	3.86	1.11	1.40	0.74	1.10	0.97	0.000
Osteoblasts	33.29	16.52	29.50	6.03	25.90	6.98	22.60	12.74	0.263

**Figure 7:**
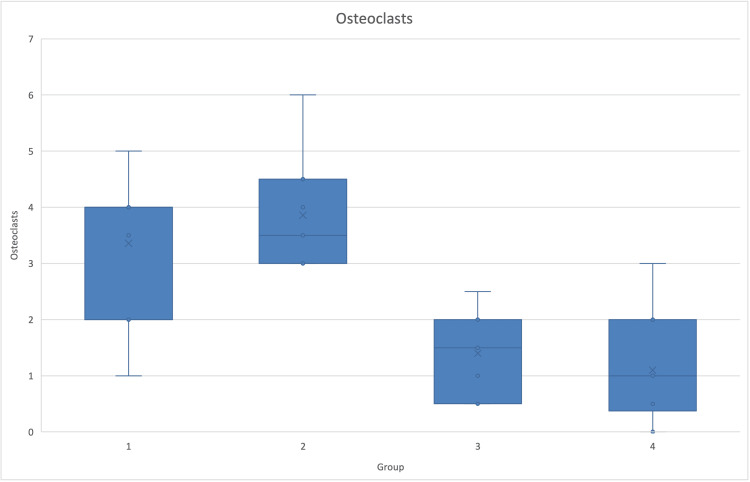
Osteoclast number per group Graphic of osteoclast numbers and SD in the four different groups. Osteoclasts were significantly lower in groups treated with incretin-based drugs (p=0.000).

There was a statistical difference in the number of osteoclasts (p<0.001, ANOVA, Tukey post hoc test) among the sitagliptin and exenatide groups compared to the control groups (Figure 7).

## Discussion

Type 2 diabetes mellitus is increasingly common in developed countries because of population growth, aging, urbanization, and the increasing prevalence of obesity and physical inactivity. Clinical studies have repeatedly shown that type 2 diabetes increases fracture risk regardless of normal or even high bone mineral density [18]. The risk is increased from poor bone quality related to prolonged hyperglycemia and also because of the increased risk of falling due to complications such as neuropathy, vascular disease, and impaired vision. Associated myopathy and hypoglycemia also contribute to the increased risk of falls in diabetic patients [19].

Diabetes has two categories of effects on the bone, primary and secondary. On one hand, chronically increased glucose may alter bone properties through disruption of bone remodeling via osteoblasts and osteoclasts. On the other hand, hyperglycemia causes the formation of advanced glycation end-products (AGEs), which, in turn, increase cross-linking between collagen fibers, resulting in stiffer and more brittle bones [20].

As previously discussed, there are two classes of incretin-based therapies: dipeptidyl peptidase-4 inhibitors, which include sitagliptin and saxagliptin and are administered orally, and glucagon-like peptide-1 receptor agonists such as exenatide and liraglutide, that are administered subcutaneously. Both categories have been used as monotherapy or in conjunction with other drugs [21]. In this study, we chose sitagliptin and extended-release exenatide due to availability and to decrease the number of injections to the animals.

In our study, diabetic bone without treatment was stronger than that of non‐diabetic controls, as well as compared to the bone of animals taking antidiabetic treatment. This appears in conflict with clinical observations of increased fracture risk in patients with type 2 diabetes mellitus; however, it highlights the complexity of the various factors that contribute to total fracture risk. Other studies have also shown similar paradoxical results in diabetic men [22], showing that pathophysiological changes of diabetes occur in many structural levels.

This paradox of increased bending strength of the diabetic bone could be explained by the fact that a decrease in osteoclasts is associated with a coupling decrease in osteoblasts and bone formation rate, so a failure to restore osteoblasts may reduce bone turnover, as it happens with certain anti-resorptive osteoporosis treatments. This blocks bone remodeling, resulting in an "older", more mineralized, and more brittle bone [23]. In our study, both incretin-based drugs reduced bone resorption; however, they did not restore osteoblastic function, resulting in potentially less fracture-resistant bone.

The differences between the two antidiabetic drugs, although not statistically significant, show a slight edge of exenatide over sitagliptin in terms of bone strength and mineralization. As previously mentioned, exenatide is a GLP-1 receptor agonist, and sitagliptin is a DPP4 inhibitor. It has been established that DPP4 inhibitors may raise GLP-1 concentrations but decrease net GLP-1 secretion [24]. This may adversely affect bone because GLP-1 has been shown to promote osteogenic differentiation while blocking adipogenic differentiation in vitro [25]. In vivo studies have shown that exendin-4, a GLP-1 receptor activator increases bone mineral density in type 2 diabetic rats [26].

Moreover, DPP4 has been identified as a potential osteoclast-derived protein with a possible role in osteoclast-osteoblast coupling but also as a potential link between RANKL/bone remodeling and energy metabolism [27]. Thus, DPP4 inhibition may decrease bone remodeling resulting in a more brittle and less fracture-resistant bone. A high serum concentration of DPP4 has been correlated with increased bone remodeling in postmenopausal women [28].

Another point to be made from our data is that relatively short-term studies may not fully reflect the secondary effects of hyperglycemia on the bone. Bone remodeling is a lengthy procedure, and considering that mineralization can last for years, this can be a possible explanation for the controversy arising from many studies that show an increased bone mass in diabetic patients. Diabetes increases bone resorption, activating bone remodeling pathways and resulting in newer and less mineralized bone. However, in the long term, this new bone gets oxidized and becomes more brittle. Studies have shown that hyper-mineralized bone tissue is more brittle because of long-term inhibition of bone turnover and reduced bone renewal rate [29].

This study has several limitations. Probably due to the short duration of the experiment, the effect of antidiabetic therapy has been obscured by the mineralization lag. Another possible confounding factor is that alterations in bone turnover do not only increase the average tissue degree of mineralization but also the variability of tissue degree of mineralization. Local variability of tissue degree of mineralization is an important contributor to bone mechanical properties, despite literature mainly focusing on the biomechanical effects of average tissue degree of mineralization [30].

## Conclusions

In summary, bone complications of type 2 diabetes occur in multiple structural levels that can be hard to control simultaneously and are tightly interwoven. Although there has been a shift in the focus of the literature from bone quantity to bone quality, there are many factors of bone architecture that contribute to net fracture risk. In our study, we found that diabetes can increase bone strength in the short term, which explains the controversy of increased bone mineral density in diabetic subjects, often found in other studies. It is, however, clear that bone remodeling balance is skewed towards bone resorption in diabetes, an effect that is only partially reversed by antidiabetic treatment. This chronic imbalance may be the key to ultimately decreased bone strength. Moreover, our study highlighted the differences between two antidiabetic treatment options, pointing towards the direction of previous correlations of DPP4 receptors with osteoblast-osteoclast coupling, although the differences were not statistically significant.

In the future, it would be interesting to see longer-term studies of the effects of diabetes on bone strength and bone mineralization to see how mineralization lag plays its role and how long it takes for the diabetic bone to reach an equilibrium, with or without antidiabetic treatment. Another potential area for further study is the effect of incretin-based drugs on local variability of bone mineralization and the subsequent results on bone strength. The clinical application of this information is not yet possible because the definite underlying mechanisms are challenging to assess individually in each patient. The onset of diabetes is gradual and not so well defined in human subjects, and various co-morbidities influence each other, so this area of study still has a lot to be explored.
